# Visual processing: conscious until proven otherwise

**DOI:** 10.1098/rsos.171783

**Published:** 2018-01-31

**Authors:** Tarryn Balsdon, Colin W. G. Clifford

**Affiliations:** School of Psychology, UNSW Sydney, Sydney, New South Wales, Australia

**Keywords:** visual perception, consciousness, awareness, signal detection theory, backward masking, computational modelling

## Abstract

Unconscious perception, or perception without awareness, describes a situation where an observer's behaviour is influenced by a stimulus of which they have no phenomenal awareness. Perception without awareness is often claimed on the basis of a difference in thresholds for tasks that do and do not require awareness, for example, detecting the stimulus (requiring awareness) and making accurate judgements about the stimulus (based on unconscious processing). Although a difference in thresholds would be expected if perceptual evidence were processed without awareness, such a difference does not necessitate that this is actually occurring: a difference in thresholds can also arise from response bias, or through task differences. Here we ask instead whether the pattern of performance could be obtained if the observer were aware of the evidence used in making their decisions. A backwards masking paradigm was designed using digits as target stimuli, with difficulty controlled by the time between target and mask. Performance was measured over three tasks: detection, graphic discrimination and semantic discrimination. Despite finding significant differences in thresholds measured using proportion correct, and in observer sensitivity, modelling suggests that these differences were not the result of perception without awareness. That is, the observer was not relying solely on unconscious information to make decisions.

## 1. Introduction

The brain does a lot of work that goes unacknowledged by conscious awareness. For instance, when we adjust the vergence of our eyes to bring into focus objects at different distances from ourselves, we are rarely aware of the small movement of the eyes, nor of the complex trigonometry that would allow for this correction. This is as unremarkable as the fact that we are blissfully unaware of the everyday affairs of our kidneys. There are some processes that should stay behind the scenes. But perception does not seem like one of those processes. Introspection would lead us to believe that we have a phenomenal experience of everything we perceive; yet experimental evidence suggests the contrary. Perceptual processing of a stimulus can occur even when the phenomenal visibility of that stimulus is limited to the point where the observer denies seeing it [1–3]. Perception without awareness implies that there is a difference between conscious and unconscious perceptual processing, and this dissociation can provide a means of examining what happens in the brain to make us conscious [4–6]. However, in order to examine the neural mechanisms of consciousness in this manner, first we must establish accurate measures of perception without awareness.

The development of techniques that halt perceptual processing revealed many interesting dissociations between conscious experience and information that is processed unconsciously [1]. One such technique is backwards masking. In backwards masking experiments, an observer is presented with a target stimulus, followed after a variable time (stimulus onset asynchrony, SOA) by a ‘mask’, a stimulus that covers the target stimulus. At short SOAs (less than about 50 ms) the observer's ability to make accurate decisions about the target stimulus is limited [7]. It is reasoned that the presentation of the mask prevents further processing of the target, such that the SOA can be manipulated to understand the temporal window necessary for the processing of different perceptual tasks [8].

A series of experiments by Marcel [9] challenged the idea that the same information available for conscious representation is used to make conscious decisions. In a backwards masking experiment, observers were shown words (or blanks) followed so quickly by a mask that they were unable to accurately judge whether a word had been presented or not. However, at those same SOAs, observers could accurately decide which of two words were more similar to the masked word in meaning (semantic discrimination judgement) and, to a lesser extent, shape (graphic discrimination judgement). It was also found that masked words could affect reaction times to subsequently presented stimuli: observers were quicker to name a colour patch if it was preceded by a masked word correctly naming the colour patch, but slower when the word named a different colour, even though observers denied seeing the masked word. These findings suggested that information unavailable to consciousness could indeed be incorporated into conscious decision making and affect behaviour, and that phenomenal awareness of stimuli may require more perceptual processing than the processing for certain decisions, such as semantic judgements.

Marcel's demonstrations of unconscious perception, and many others since, rely on a difference in performance thresholds for awareness of the stimulus and another perceptual task. Typically, the threshold for awareness is measured using a detection threshold in a separate task where the target stimulus is only presented on half the trials and the observer must judge whether or not it was present [9–11]. Alternatively, in more recent experiments, the observer is simply asked for a subjective report as to whether they saw the stimulus on each trial of the perceptual task [12–14]. The threshold is calculated from the detection task as the point at which half the observer's correct responses can be attributable to guessing (75% correct) or, from subjective reports as 50% ‘seen’. This threshold is then compared to performance in a separate perceptual task, such as semantic discrimination, in which a second threshold is calculated in a similar manner. A difference in thresholds, where the threshold for awareness of target presence is greater than the threshold in the perceptual discrimination task, is taken as evidence for perception without awareness, as it is reasoned that accurate performance below the threshold for awareness must rely on unconscious perceptual evidence. However, this difference in thresholds does not unequivocally demonstrate perception without awareness; although a difference in thresholds would be expected if perceptual evidence were being processed without reaching awareness, the difference may have arisen for a number of other reasons.

The first problem with the approach of looking for a difference in thresholds is selecting which thresholds to compare. Many different measures of thresholds have been used in the literature, with the choice of threshold measure having a significant impact on experimental results. Marcel [9] found lower thresholds for semantic compared to graphic discrimination. Similar results have been found for ‘low-level’ and ‘high-level’ tasks; in ‘high-level’ tasks observers tend to show lower thresholds than in ‘low-level’ tasks. For example, observers can better discriminate consonants from vowels (a ‘high-level’ task) compared to discriminating upper from lower case letters (a ‘low-level’ task) [15]. Observers have also been shown to better discriminate whether Arabic digits are greater or less than 5 (a ‘high-level’ task) compared to making a colour discrimination (a ‘low-level’ task; [16]). These differences in performance suggest that the type of perceptual task used to measure unconscious processing of perceptual information is important, as different tasks can be performed to different extents on the basis of unconscious evidence. If the task chosen to measure the threshold of unconscious perception were too difficult, evidence for perception without awareness may not be found at all.

Perhaps an even more influential factor in whether evidence for perception without awareness is found is the measurement of the threshold for awareness. For example, Cheeseman & Merikle [17] found no evidence for unconscious processing of semantic information below an objectively defined threshold for awareness (observers' discrimination thresholds) in a colour priming task, where a briefly presented colour word is expected to influence reaction times to a subsequently presented colour patch. However, in a later study, they did find priming effects below a subjectively defined threshold for awareness—the SOA at which observers reported they saw the stimulus less than 55% of the time (Cheesman & Merikle [18]). To some extent the choice of threshold for awareness is definitional. Some argue that consciousness is such a subjective phenomenon that it requires a subjective measure [19–21], while others argue for more objective thresholds [22–24], the most conservative rationale (offered by Macmillan [25]) being that if the observer really had no awareness of the stimulus then they should make as many false alarms (when the observer reports the stimulus as being present when it is not) as they do correct detection decisions, which corresponds to no sensitivity to the stimulus at all (*d*′ = 0) under signal detection theory (SDT [26]).

However, the problem of selecting the correct threshold measure is not just definitional. One must also take into account how the measured threshold might be influenced by non-perceptual processes, such as the observer's willingness to make a certain response. For instance, if using subjective measures of awareness, such as the perceptual awareness scale (PAS; [13,27]) or reports of whether the observer saw the stimulus or not, it is possible that observers might be biased against reporting that they saw the stimulus at noticeably shorter SOAs, even though they might have had some awareness of the stimulus. Furthermore, subjective measures leave the definition of ‘aware’ up to the observer; while one observer might only report that they were aware of the stimulus when they were absolutely certain they saw it, another might report that they were aware when they thought they only saw a brief flash. It has even been shown that observers can adopt different response biases depending on the wording of the instructions for reporting subjective awareness [28]. These problems reflect a general problem of response bias in subjective measures, which corrupts the accuracy of the measure, and can lead to exaggerated differences between perception and awareness [29]. Given that observers might also be less likely to adopt a biased response strategy in the objective task used to measure their unconscious processing threshold, the comparison of thresholds also becomes unfair, since bias always reduces proportion correct performance, as any deviation from the optimal criterion results in a net increase in incorrect responses [30].

The problem of response bias can be reduced through the use of SDT [26] to calculate the observer's sensitivity to the stimulus (*d*′) irrespective of their willingness to make a particular response (their criterion, c). However, there is no specific framework in SDT for defining a threshold [30]. One could take an arbitrary value of *d*′ as a threshold, such as *d*′ = 1, which would correspond to approximately 76% correct (unbiased) in a task where two stimuli are presented in equal proportions, but when it comes to a threshold for awareness, there is no value of *d*′ by which the theoretical foundation suggests that an observer is not aware. On the contrary, Macmillian [25] justifies five different possible definitions of ‘threshold’ for awareness in SDT terms.

To avoid the threshold problem, one could simply compare *d*′ over a range of SOAs to test whether there are significant differences in the sensitivity of observers between tasks. However, in this case one also needs to be careful of avoiding any expected differences due to the task. For example, it is well documented that a difference in sensitivity should be expected between single- (yes/no) and two-interval (forced choice) tasks [30]. Differences in sensitivity are also frequently observed between identification and detection tasks [31], and even classification compared to discrimination tasks [32]. However, each of these differences in measured sensitivity has been explained by a difference in task demands, rather than an actual difference in perceptual sensitivity to the stimulus (e.g. [33–35]). Thus, when comparing performance across two tasks as evidence for perception without awareness, one also needs to accommodate for potential differences in task demands that may appear as differences in sensitivity between the two tasks.

A related problem with comparing measures across tasks comes from considering what information observers are using to make their responses, or their ‘criterion content’ [36]. The method of backwards masking is especially vulnerable to this limitation because the manipulation of ‘visibility’ is frequently accompanied by changes to the interaction between target and mask. That is, at shorter SOAs the target may become integrated with the mask and thereby become more visible than at medium duration SOAs [28], or the transition from target to mask may cause a perception of motion [37]. This means that the observer may use different information at different SOAs and so the comparison between tasks across SOAs may actually compare decisions based on different evidence. The same level of performance could then be achieved by a single observer using two different sources of visual evidence to make their decisions. Thus it is important not only to consider how observers are making decisions but also what information they are basing their decisions on and whether this could change with experimental manipulations.

Here, we test a new approach for examining perception without awareness. Instead of taking a mere difference in thresholds or a difference in sensitivity as evidence, we ask whether it is possible that the observer's pattern of performance could have been obtained if the observer was only using conscious evidence, that is, without relying on unconscious evidence to make their decisions [38]. To test this, we implement a family of models, whereby the observer is only able to decide correctly if they consciously ‘saw’ the stimulus, otherwise they guess. Thus the basic model requires just a ‘rate of seeing’ and a ‘criterion for guessing’. The ‘rate of seeing’ is assumed to increase with increasing stimulus evidence/processing (such as with a longer SOA between target and mask) in the form of a Weibull function [39]. The model fit to the hit and false alarm rate data takes a similar form as a high threshold model (as discussed in [30]), where false alarms only arise from guessing.

The ‘conscious’ family of models described above assumes the observers' judgements are based on the same evidence (the ‘rate of seeing’). To test whether this is a good description of the data, we also compared the fit of the models to a comparable family of models that allowed detection performance to be based on different evidence to the semantic and graphic discrimination tasks. This new family of models, the ‘unconscious’ models, simulates a situation where the discrimination judgements may be based on unconscious evidence, while the detection judgements must wait for perceptual processing for awareness. The ‘unconscious’ models are the more complex, as additional parameters are required to describe the unconscious evidence. This is advantageous as it means the ‘unconscious’ model can be treated as an alternative hypothesis (there must be sufficient evidence to justify the more complex model). In contrast, other analyses for showing unconscious processing often encounter the statistical problem of corroborating the null, for example, by showing that detection *d*′ is not significantly different from zero [40].

The models were fit to the hit and false alarm rate data from five observers in a backwards masking experiment based on previous methodologies in the literature [9,10,41,42]. Target stimuli were the Arabic digits 1, 3, 7 and 9, and were masked after a variable SOA by the letters O, T and X superimposed over one another. In separate blocks, participants were asked to complete three separate tasks. Two were perceptual tasks that previous evidence suggests can be mediated by unconscious perception: a semantic discrimination task, where observers were asked whether the target stimulus was greater or less than 5, and a graphic discrimination task, where observers were asked if the number was more round or straight in shape. The numbers were balanced such that in the semantic task the numbers less than 5 were round and straight and in the graphic task the round numbers were both greater and less than 5, and vice versa. The third task was a detection task, which is assumed to require awareness of the target stimuli. In the detection task, unlike the other tasks, the target stimuli were presented on only half the trials, and the other half were blank; observers were asked to decide whether or not a target stimulus had been presented. The objective detection task was chosen as a measure of awareness over subjective rating methods so that *d*′ can be compared across all tasks. Furthermore, previous evidence has shown that in practised observers subjective ratings closely follow detection performance [43].

The analysis aims at comparing the proportion correct threshold method of establishing perception without awareness with the bias free SDT measures and the more conservative method of testing whether the pattern of performance could be obtained if the observer only used perceptual evidence of which they were aware. If the difference in proportion correct thresholds can be explained by the model assuming only conscious evidence was used to make responses then the data do not provide evidence of perception without awareness.

## 2. Methods and materials

### 2.1. Participants

Five experienced observers (two authors) gave written informed consent to participate after the experimental procedures were explained to them. All were right handed, with normal or corrected to normal vision. Ethical approval was granted by the UNSW human research committee, which adheres to the declaration of Helsinki.

### 2.2. Stimuli

**Figure 1.**
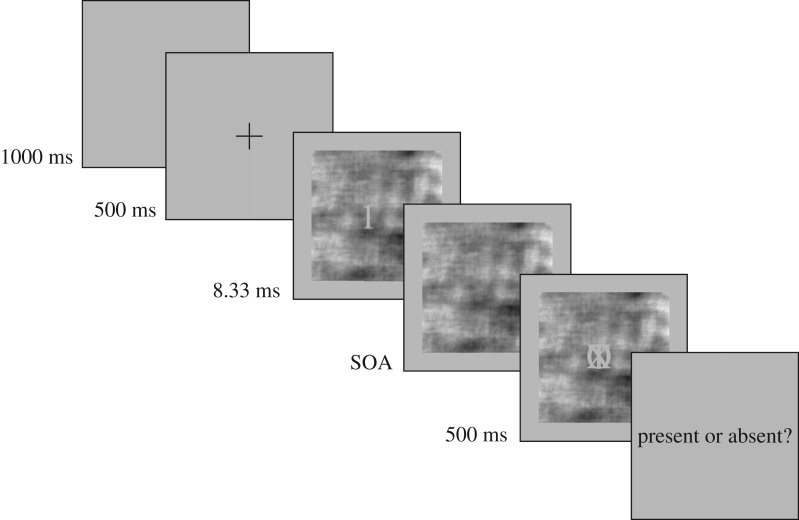
Example stimulus presentation. Only the centre 256 × 256 pixels and surround is shown. The target stimulus is presented over a patch of noise for 8.33 ms, the noise patch remained during the variable SOA, then the mask is presented for 500 ms, followed by the response prompt.

Stimuli were displayed on a 32^″^ Display++ LCD monitor (Cambridge Research Systems, Rochester, UK) with a refresh rate of 120 Hz and resolution 1920 × 1080, with a grey background, mean luminance 60 cd m^−2^. The four target stimuli were Arabic digits (1, 3, 7 and 9) and were displayed in Calibri font in grey such that they subtended 5° of visual angle. The mask stimulus was composed of the letters O, T and X, superimposed over one another, also in Calibri font in grey and subtending 5° of visual angle. The start of each trial was cued with a fixation cross for 500 ms. The target digits were presented in the centre of a 256 × 256 pixel (approximately 9° of visual angle) patch of random noise, in the centre of the screen. The noise patch was filtered to have the same spatial amplitude spectrum as the mean spatial amplitude spectrum of the digits themselves, and with the same mean luminance, and root mean squared contrast of 0.15. Target stimuli were displayed for 8.33 ms (with luminance 60 cd m^−2^), and after a variable SOA, were followed by the mask (presented at the same luminance), the noise patch remained on screen throughout the trial. The position of the mask was jittered slightly from trial to trial to prevent observers learning to look in a particular location where the mask might be weaker, and prevent any integration between target and mask from providing reliable cues to the identity of the target. The mask was displayed for 500 ms before the observer was prompted to respond. In the detection experiment, 50% of trials were ‘target absent’ trials, in which no target stimulus was presented, only the noise patch. An example ‘target present’ trial from the detection task is displayed in figure 1. Stimulus presentation was coded in Python using the PsychoPy library [44,45]. Stimulus timings were carefully monitored by recording the screen flip interval timings on each trial. There were no trials in which flips were missed.

### 2.3. Procedure

Throughout the experiment, observers sat approximately 57 cm from the screen. Observers completed three tasks: detection, semantic discrimination and graphic discrimination. Each task was split into three blocks, the order of which was pseudo-randomized between participants. Most participants completed the experiment over three 1 h sessions, each containing one block of each task. In each task, 96 trials were completed at each SOA. The SOAs for each observer were defined using a short practice block of the detection task (16 trials at a range of SOAs) prior to commencement of the experiment, these trials also served as practice. For each observer, SOAs were chosen such that performance in the detection task would range from chance to ceiling, with as many SOAs in between as time would allow for. For three observers SOAs of 16.7, 25, 33.3, 41.7, 50, 66.6 and 83.3 ms were used, one observer was given SOAs of 8.3, 16.7, 25, 33.3, 41.7, 50 and 66.6 ms and the remaining observer was given SOAs of 16.7, 33.3, 50, 66.6, 83.3 and 100 ms. These SOAs were used in all three tasks.

In all three tasks the stimuli and response keys were the same, all that changed were the instructions given to the observer, with the exception that in the detection task, no target stimulus was presented on half the trials (target absent trials). In the detection task observers were instructed to press ‘1’ if a target stimulus was presented and ‘2’ if no target stimulus was presented. In the semantic discrimination task observers were instructed to press ‘1’ if the target stimulus was a number less than 5, or ‘2’ if the target stimulus was a number greater than 5. In the graphic discrimination task, observers were instructed to press ‘1’ if the target stimulus was straight in shape, and ‘2’ if the target stimulus was round in shape. Observers were cued for the response after each trial with the words ‘present or absent?’ in the detection task, ‘less or greater than?’ in the semantic discrimination task, and ‘straight or round?’ in the graphic discrimination task.

Immediately after their response they were also asked to give a pseudo-type 1 confidence rating [46] ranging from 1 (low confidence) to 4 (high confidence). The pseudo-type 1 rating is defined as confidence in the response, for example, in the detection task, a rating of 3 following a ‘target present’ response indicates ‘moderately high confidence that the target was present’, whereas a rating of 2 following a ‘target absent’ response indicates ‘moderately low confidence that the target was absent’. Note that these are essentially type 1 ratings, used for estimating the receiver operating characteristic (ROC) curve, not the type 2 ratings used in metacognition research (see [43]). Observers were instructed to try to use the whole range of confidence ratings. Observers entered these ratings with their left hand on the top set of numbers on a standard keyboard, while the discrimination responses were entered with their right hand on the right set of numbers on the keyboard. The rating response cued the start of the next trial (the experiment would halt until a response was entered), which began with a 1000 ms inter-trial interval.

### 2.4. Analysis

There are three parts to the analysis: a comparison of proportion correct thresholds, a signal detection theory (SDT) analysis, and modelling. In each case, the analysis was conducted within subjects.

#### 2.4.1. Proportion correct

The analysis of proportion correct was conducted in the usual way, by taking the number of correct responses (for each SOA, for each task) and dividing by the total number of responses. Chance performance is taken as a proportion correct of 0.5, and ceiling performance at 1. Proportion correct measurements are vulnerable to response bias, where, for example, an observer who is unwilling to respond that a stimulus was present (when evidence is weak) will score a lower proportion correct compared to an observer who is willing to guess that a stimulus was present based on weak evidence.

#### 2.4.2. Signal detection theory analysis

Observer responses in combination with their ratings were transformed into an 8-point rating scale, and sensitivity (*d*_a_) was calculated using RscorePlus (v. 5.6.1; [47]), which performs a maximum-likelihood fit of ROC curve parameters to each point on the curve estimated by the confidence ratings data, assuming a Gaussian model of additive sensory noise. *d*_a_ was taken as a measure of sensitivity as it does not assume equal variance between the noise only and signal-plus-noise distributions and is numerically identical to *d*′ when the equal variance assumption holds [48].

#### 2.4.3. Modelling

A simple (‘conscious’) model with two parameters was designed to test whether any differences in detection and discrimination sensitivity could be explained by a difference in task demands alone. These parameters fit a rate of ‘seeing’ as a Weibull function, with alpha as the midpoint position parameter and beta as the slope:

$$s(x)=1-{\text{e}}^{-{\left(x/\alpha \right)}^{\beta }},$$

where *s* is the proportion of stimuli ‘seen’ at each SOA (*x*). In the case of discrimination performance HR (Hit Rate) and FAR (False Alarm Rate) are then calculated as:

$$\begin{array}{rl} \text{HR} & =s(x)+c\times (1-s(x))\\ \;\text{and}\;\text{FAR} & =c\times (1-s(x)), \end{array}$$

where *c* corresponds to the criterion for guessing (where unspecified, is set to 0.5). For detection, HR and FAR are calculated as:

$$\begin{array}{rl} \text{HR} & =s(x)+f\times (1-s(x))\\ \;\text{and}\;\text{FAR} & =f, \end{array}$$

where *f* is simply the false alarm rate (the proportion of times the observer will report that the target was present when it is actually not). This difference in the false alarm rates between tasks expresses the difference in task demands: when they do not see the stimulus in the discrimination task the observer must guess, whereas, in the detection task, not seeing the stimulus could be taken as evidence that the stimulus was not present.

**Figure 2.**
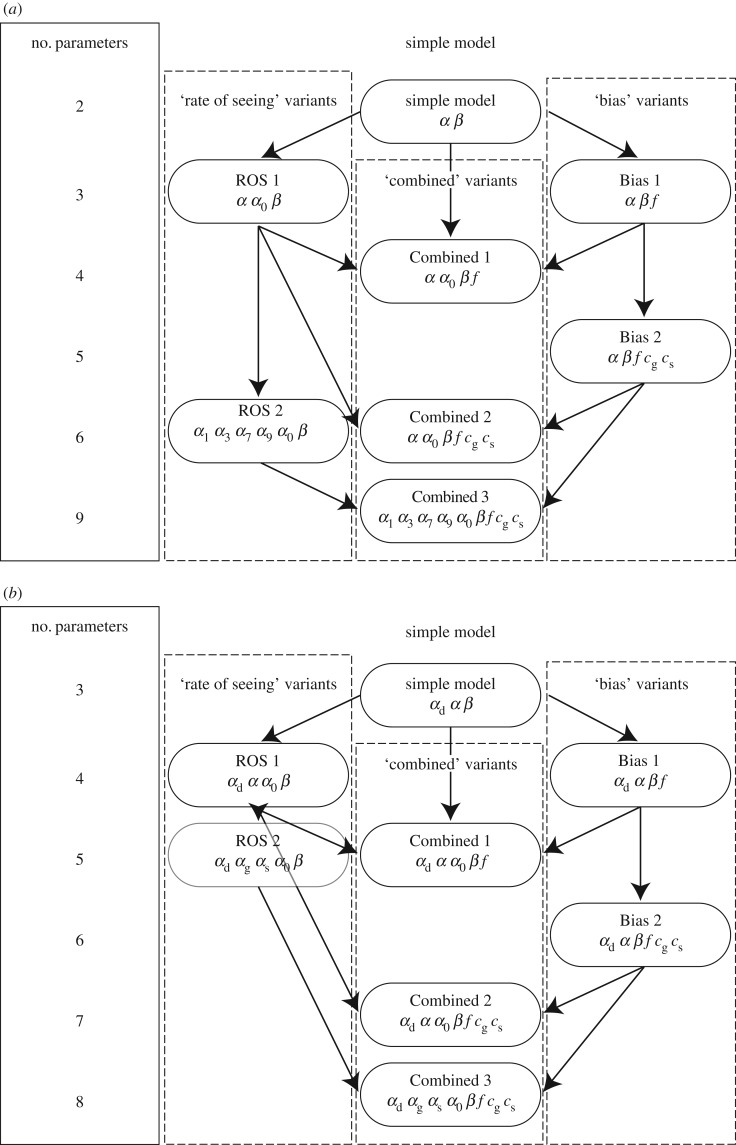
Graphical representation of the conscious model family (*a*) and the analogous unconscious model family (*b*). Arrows linking each model to more complex models shows how models are built upon to create more complex models. Each *α* corresponds to the midpoint position parameter of one rate of seeing, while the same slope parameter, *β*, was used for each rate of seeing. The parameters *f* and *c* refer to criterion parameters for the detection and discrimination decisions respectively.

From the simple ‘conscious’ model, two families of more complex models could be defined (figure 2). One successively adds midpoint parameters (*α*) such that differences in performance could be further explained by the different visibilities of the stimuli (henceforth described as rate of seeing, ROS models). The other successively adds criterion parameters such that difference in performance could be further explained by different biases in responding in different tasks (henceforth described as Bias models). ROS 1 adds a second rate of seeing midpoint, *α*_0_, corresponding to the rate at which an observer sees that no stimulus was present (such that the detection $\text{FAR}=f\times (1-s_{0})$). ROS 2 adds different midpoints for rates of seeing to each stimulus (with a total of five intercepts for rates of seeing). Bias 1 adds a parameter for the false alarm rate in the detection decision (so that *f* may differ from 0.5) and Bias 2 adds two more parameters for criteria in the semantic and graphic decisions separately. Finally, all parameters could be combined to give even more complex models (henceforth described as Combined models). Three of these are tested; Combined model 1 adds the parameters of ROS 1 and Bias 1, Combined 2 adds the parameters of ROS 1 and Bias 2, and Combined 3 adds parameters from ROS 2 and Bias 2. The full equations are written in table 1.

**Table 1.** Equations for fitting the hit and false alarm rates under each model. Letters *c* and *f* refer to fit ‘guess rate’ parameters, and *s* refers to the ‘rate of seeing’, which is a function of the SOA defined by two parameters, *α* and *β*. Multiple subscripts refer to multiple *α* to define different rates of seeing, where a single *β* was fit for all *s* (where subscripts *s* and *g* refer to semantic and graphic discrimination respectively, and *a*–*d* refer to each target stimulus—1, 3, 7 and 9, respectively, for the semantic discrimination task, and 1, 7, 3 and 9, respectively, for the graphic discrimination task). The ‘conscious’ columns contain equations for models assuming the same evidence is used for discrimination and detection decisions, while the ‘unconscious’ columns contain equations where different evidence is used.

	conscious		unconscious	
	discrimination	detection	discrimination	detection
Simple				
HR	*s* + 0.5 × (1 − *s*)	*s* + 0.5 × (1 − *s*)	*s* + 0.5 × (1 − *s*)	$s_{d}+0.5\times (1-s_{d})$
FAR	0.5 × (1 − *s*)	0.5	0.5 × (1 − *s*)	0.5
ROS 1				
HR	*s* + 0.5 × (1 − *s*)	*s* + 0.5 × (1 − *s*)	*s* + 0.5 × (1 − *s*)	$s_{d}+0.5\times (1-s_{d})$
FAR	0.5 × (1 − *s*)	0.5 × (1 − *s*_0_)	0.5 × (1 − *s*)	0.5 × (1 − *s*_0_)
ROS 2				
HR	s+0.5×(1−s) where $s=(s_{a}+s_{b})/2$	*s* + 0.5 × (1 − *s*) where $s=\;(s_{a}+s_{b}+\;s_{c}+\;s_{d})/2$	$s_{g/s}+0.5\times (1-s_{g/s})$	$s_{d}+0.5\times (1-s_{d})$
FAR	0.5 × (1 − *s*) where $s=(s_{c}+s_{d})/2$	0.5 × (1 − *s*_0_)	$0.5\times (1-s_{g/s})$	0.5 × (1 − *s*_0_)
Bias 1				
HR	s+0.5×(1−s)	s+f×(1−s)	s+0.5×(1−s)	$s_{d}+f\times (1-s_{d})$
FAR	0.5 × (1 − *s*)	*f*	0.5 × (1 − *s*)	*f*
Bias 2				
HR	$s+c_{g/s}\times (1-s)$	s+f×(1−s)	$s+c_{g/s}\times (1-s)$	$s_{d}+f\times (1-s_{d})$
FAR	$c_{g/s}\times (1-s)$	*f*	$c_{g/s}\times (1-s)$	*f*
Combined 1				
HR	*s* + 0.5 × (1 − *s*)	s+f×(1−s)	*s* + 0.5 × (1 − *s*)	$s_{d}+f\times (1-s_{d})$
FAR	0.5 × (1 − *s*)	$f\;\times (1-\;s_{0})$	0.5 × (1 − *s*)	$f\;\times (1-\;s_{0})$
Combined 2				
HR	$s+c_{g/s}\times (1-s)$	s+f×(1−s)	$s+c_{g/s}\times (1-s)$	$s_{d}+f\times (1-s_{d})$
FAR	$c_{g/s}\times (1-s)$	$f\;\times (1-\;s_{0})$	$c_{g/s}\times (1-s)$	$f\;\times (1-\;s_{0})$
Combined 3				
HR	s+c×(1−s) where $s=(s_{a}+s_{b})/2$	s+f×(1−s) where $s=\;(s_{a}+s_{b}+\;s_{c}+\;s_{d})/2$	$s_{g/s}+c_{g/s}\times (1-s_{g/s})$	$s_{d}+f\times (1-s_{d})$
FAR	c×(1−s) where $s=(s_{c}+s_{d})/2$	$f\;\times (1-\;s_{0})$	$c_{g/s}\times (1-s_{g/s})$	$f\;\times (1-\;s_{0})$

These ‘conscious’ models were then compared to corresponding ‘unconscious’ models that allowed the evidence used in the detection and discrimination tasks to differ, by adding a separate midpoint parameter to describe the ‘rate of seeing’ the targets in the detection task. Theoretically these models allow the rate of seeing that constrains performance in the detection task to differ from the rate of potentially unconscious evidence accumulation that constrains the discrimination tasks. For ROS 2 and Combined 3, instead of adding separate midpoint parameters for seeing each stimulus, it was simpler to use separate parameters for each task, since the parameters for each stimulus would be averaged into a single rate of seeing prior to comparing the model predictions to the observed data (table 1).

The best fit of each model for each observer was found by minimizing the sum of squared error (SSE) between the predicted HR and FAR and the HR and FAR obtained at each SOA. For each observer, the simplest model was fit first with the same starting values for each observer, the parameters were then adjusted to find those minimizing the difference between the actual and predicted data using the simplex search method [49]. Proceeding down the family tree of models, the parameters from the previous model were used as starting parameters for the more complex models where possible. For the unconscious models to be a better description of the data, there needs to be enough of a difference between the detection and discrimination tasks to require that they be described with different rates of evidence accumulation.

## 3. Results

### 3.1. Proportion correct

**Figure 3.**
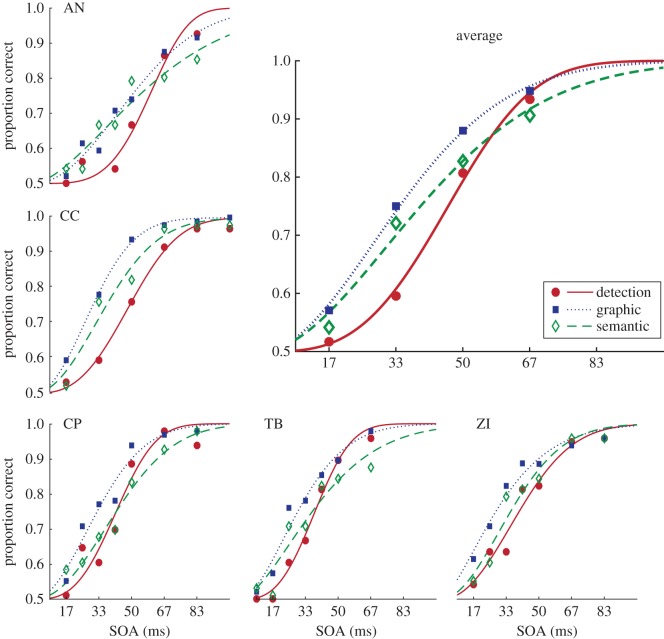
Proportion correct scores in each task for each participant. Each participant's scores in the detection (circle symbols), semantic discrimination (diamonds) and the graphic discrimination (squares) tasks are shown with the best fitting Weibull function. The top right panel shows the data averaged across the five participants, for the four SOAs that all participants were tested on, to show the general trend.

Each participant showed a greater proportion correct in the discrimination tasks than the detection task at critical SOAs (where performance was above chance and below typical definitions of threshold). The typical pattern was for best performance in the graphic discrimination task, followed by the semantic discrimination task and finally the detection task, as shown in figure 3.

### 3.2. Signal detection theory analysis

**Figure 4.**
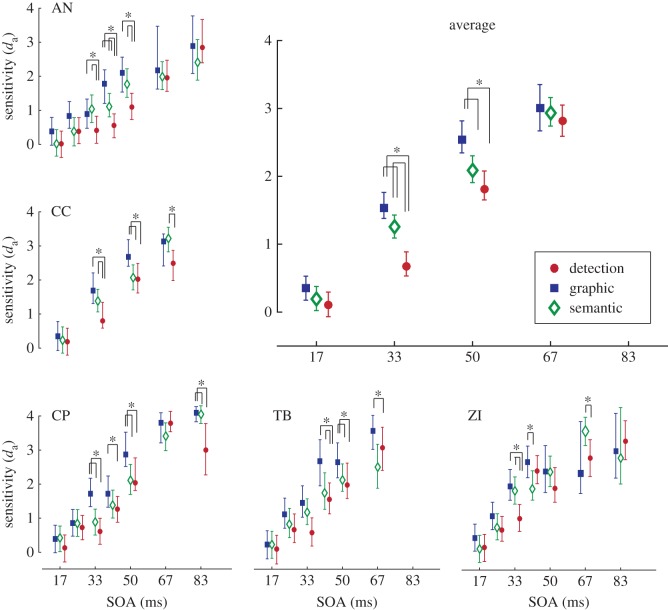
Sensitivity (*d*_a_) in each task across each SOA tested. Error bars mark the 95% confidence interval based on 1000 bootstraps of the data. The top right panel shows the average *d*_a_ across the four SOAs that all participants were tested on, error bars here represent 95% confidence intervals from an average of the bootstraps. Asterisks mark where less than 5% of the bootstrapped data overlap between tasks.

The pattern for *d*_a_ was similar to that for accuracy, with greatest sensitivity in the graphic discrimination task, followed by the semantic discrimination task, and finally the detection task. To assess whether these differences could be described as statistically significant, 95% confidence intervals were calculated by bootstrapping the data. For each participant, for each task, at each SOA, a random selection of their responses were taken (with replacement) to match the number of responses in the actual experiment, and *d*_a_ was calculated. This was repeated 1000 times, and the 95% confidence interval was defined by the *d*_a_ that was 25th from the smallest and the *d*_a_ that was 25th from the greatest. To compare across tasks, we took the proportion of times out of 1000 sensitivity in the detection task was greater than in the semantic or graphic discrimination tasks (and when sensitivity in the semantic discrimination task was greater than in the graphic discrimination task), proportions smaller than 0.05 were taken to be significant. The average plot in figure 4 shows significant differences in performance between the detection and discrimination tasks at 33 and 50 ms, and similar differences are visible in individual observers within the same 33–50 ms window.

### 3.3. Modelling

With each additional parameter, the proportion of variance explained (calculated as the difference between the sum of squared error and the total variance, divided by the total variance) increased; however, this is expected, especially when the models are nested. The average proportion of variance explained by each model is displayed in table 2. To properly compare the models, given the differences in the number of parameters, the Akaike Information Criterion [50] with a correction for small samples (AIC_c_; [51]) was calculated by the formula:

$$\text{AI}{\text{C}}_{c}=2k+n\times \ln(\text{SSE})+2k\times \left(\frac{n}{n-k-1}\right),$$

where *k* is the number of parameters (plus 1 for the error term) and *n* is the number of data points fitted. The AIC_c_ values, averaged over observers' individual AIC_c_ values are shown in table 3, the smaller the AIC_c_, the better the fit. For each observer, ‘conscious combined 3’ was the best fitting model, with the smallest AIC_c_, and a difference greater than 10 from both the next most complex model in the ‘conscious’ tree, and the ‘unconscious combined 3’ model, suggesting strong evidence for preferring the ‘conscious combined 3’ model. When comparing other conscious models with their unconscious analogues no unconscious model was consistently strongly preferred based on individual AIC; examining the averages, there was little evidence in favour of the unconscious models.

**Table 2.** Proportion of variance explained by each model in the conscious and unconscious families.

	conscious		unconscious	
model	no. parameters	proportion of variance	no. parameters	proportion of variance
Simple	2	0.61	3	0.64
ROS 1	3	0.66	4	0.69
ROS 2	6	0.77	5	0.70
Bias 1	3	0.71	4	0.72
Bias 2	5	0.81	6	0.83
Combined 1	4	0.76	5	0.76
Combined 2	6	0.86	7	0.86
Combined 3	9	0.93	8	0.87

**Table 3.** AIC_c_ values for each model in the conscious and unconscious families. Colours closer to green indicate lower values (and therefore better models) while more red indicates greater values (and therefore less good models). Here the number of parameters includes the error term, as used in the calculation of AIC_c_.

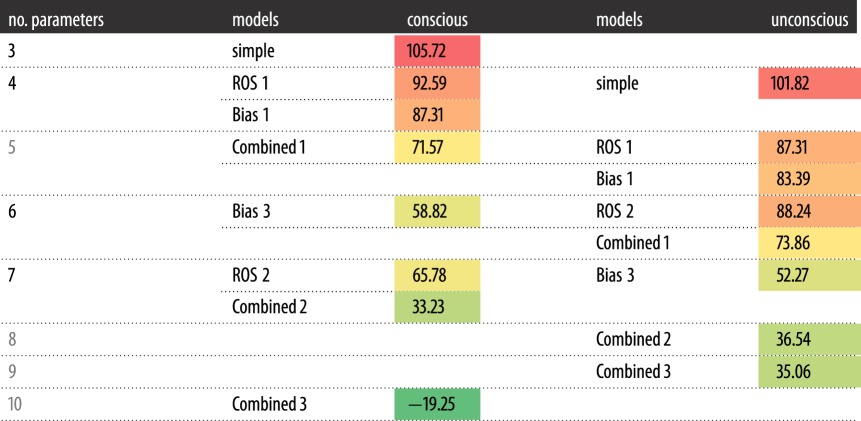

## 4. Discussion

Thresholds measured using proportion correct followed a similar pattern as expected from previous research [9,10,15,16] with lower thresholds in the discrimination tasks than in the detection task. Contrary to previous research [9,15,16], the thresholds measured in the graphic discrimination task (a ‘low-level’ task) were lower than in the semantic discrimination task (a ‘high-level’ task). We have reason to believe the differences in performance on the discrimination tasks in this experiment may actually be the result of differences in stimuli, and this explanation may generalize to other findings, this will be addressed further below. Were a difference in thresholds between detection and discrimination tasks taken as evidence for perception without awareness, we would conclude that observers were using unconscious evidence to make their responses in the discrimination tasks. However, we sought to rule out whether there could be any other explanation for the difference in thresholds, and test a new measure of performance without awareness.

First, measures of sensitivity across the three tasks were compared. It was hypothesized that a difference in detection and discrimination proportion correct thresholds could be the result of differences in response bias, where an observer may be unwilling to guess that a target was present in the detection task, thereby lowering their proportion correct compared to relatively unbiased performance in the discrimination tasks. *d*_a_ (equivalent to *d*′ when the equal variance assumption holds) measures an observer's sensitivity to the stimulus irrespective of their bias. We found that the differences in performance were still present when using bias free measures, so it is unlikely that the detection threshold was greater than the discrimination threshold simply because of differences in response bias. If an arbitrary threshold value were set, such as *d*′ = 1, these data could also be taken as evidence of perception without awareness, since across all subjects *d*′ in the discrimination tasks seemed to rise above 1 at shorter SOAs than *d*′ in the detection task. Of note, significant differences in sensitivity were found only for mid-range SOAs, where *d*′ for all tasks was significantly greater than 0; no difference was found when detection sensitivity was not significantly different from 0, only when there was some sensitivity for detecting target stimuli. If a stricter definition of unconscious perception were taken, such as detection *d*′ = 0, then these data would offer no evidence for perception without awareness.

We then fit a family of models to the data, to test whether the pattern of sensitivity could be explained without assuming unconscious evidence was being used to make responses. The models were based on the assumption that an observer could only make an accurate response if they consciously ‘saw’ the stimulus, otherwise they guessed. The winning model was able to accommodate for task differences by applying a different bias in each task and different rates of seeing each target stimulus as well as a rate of seeing that no stimulus was present in the detection task. This model explained on average 93% of the variance in the data and performed better than a model that allowed performance in each task to be based on different evidence, as would be required if the discrimination tasks were based on unconscious evidence while the detection task relied on conscious awareness. Furthermore, by comparing the AIC_c_ of the models, no unconscious model offered strong evidence for preferring it over its conscious counterpart, suggesting the data are better explained by assuming that the same evidence is used to make decisions in all the tasks.

**Figure 5.**
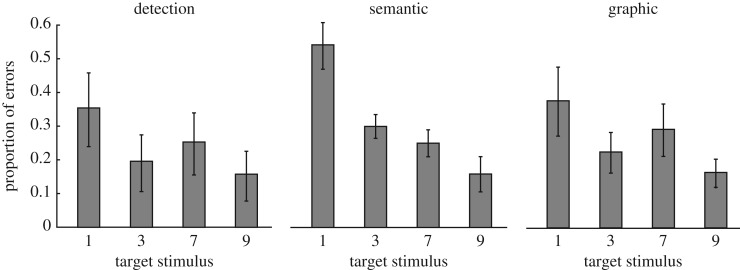
Proportion of incorrect responses to each target stimulus in each task. Proportions were averaged across observers for SOAs less than 50 ms. Error bars show one standard error of the mean.

That the same evidence was used to make decisions across all tasks not only suggests that there is nothing special about the dissociation between performance in the detection and discrimination tasks, but also that there is no difference in the evidence being used to make the semantic and graphic discrimination decisions. Specifically, differences in performance between the semantic and graphic discrimination tasks were arguably the result of either differences in response bias or differences in the processing of the individual stimuli (or, perhaps most likely, a combination of both). As can be seen from figure 5, observers made most errors when presented with the target stimulus ‘1’, and next most, ‘7’. If the processing of these stimuli was more inhibited by the mask, as is reflected by the different ‘rates of seeing’ in the winning model, then this would serve as an advantage in the graphic discrimination task, where ‘unseen’ stimuli could be accurately guessed to be straight, whereas, in the semantic task, no advantage could be had, given that ‘1’ and ‘7’ corresponded to different responses. This is problematic for theories that rely on differences in performance across discrimination tasks. In particular, the ‘level of processing’ hypothesis proposed by Windey *et al*. [16] suggests that differences in performance between a ‘high-level’ task (discriminating whether a digit was greater or less than 5) and a ‘low-level’ task (discriminating the colour of the same digit stimuli) reflect a link between the level of processing required to make a decision about a stimulus and the graded versus dichotomous nature of awareness of those stimuli. The present results suggest that the differences observed by Windey *et al*. may have simply been the result of differences in how efficiently the shape and the colour of the digit were masked. Given that the differences in the efficiency of masking for each target were noticeable from an observer's detection performance, ensuring equal detection performance across stimuli might minimize this problem.

Differences in masking between stimuli may also have occurred because of interactions between target and mask. This is problematic because it suggests that the appearance of the mask may have changed with SOA, providing different information to base responses on (criterion content) at different SOAs. Indeed, more recent theories of masking emphasize the role of integration between target and mask [52,53] and the multidimensionality of masking, where one aspect of a stimulus may be masked differently to another [54,55]. This is problematic because at short SOAs observers may be able to make accurate decisions in one task based on the appearance of the mask (which is altered by the presence of the target), but this information may not have aided another task—the comparison becomes unfair. We think it is unlikely that observers' responses were impacted by target–mask interactions in this experimental design. If there had been integration between target and mask at short SOAs, then this would have benefited performance in the detection task the most, since it should be easier to detect an artefact than to discriminate differences in artefacts, but this was not observed. If there had been an interaction between target and mask, such as an apparent motion signal, integration between target and mask or some other artefact, then this would not have remained consistent trial to trial, since the position of the mask was jittered relative to the target. Rather, it is likely that some of the stimuli were simply more effectively masked than others, just as some types of stimuli are more effectively masked by certain masking shapes [56,57].

Of note, the models we employed to examine whether observers were using the same information to make decisions across tasks also assumed that the same information was being used across SOAs. The models would therefore not accurately describe the decision making processes had there been an interaction between target and mask that changed the information used by the observer at different SOAs. The models are also limited in their application to Type B masking functions, where the masking function takes a U-shape, with performance most impaired at medium SOAs, and superior performance at short and long SOAs [58].

One limitation of this technique is that it must be conducted within observers. For this reason, testing large samples is not only time consuming (each participant must contribute a large number of trials), but means that experiments become over-powered for finding small effects. However, with smaller samples, the variability of the general population may be underestimated. For example, there is increasing evidence for genetic factors contributing to the magnitude and shape of the masking function, such as in patients with schizophrenia, and their immediate family [59]. Although small samples are unlikely to give a good estimate of this genetic variability associated with masking in general, they can be appropriate for understanding whether the technique is genuinely appropriate for dissociating conscious and unconscious processing, as we find here, observers' responses were not indicative of perception without awareness. A second, related, limitation is that many experimental procedures that are used to limit phenomenal awareness are vulnerable to a large number of parameters. For example, the magnitude and shape of masking functions can be affected by the target stimulus eccentricity, size, contrast, luminance, and the relationship of these parameters to the same parameters in the mask stimulus [60]. The specific modelling analysis conducted here is appropriate for analysing rates of seeing that can be described as monotonically increasing with some variable (in this case, the SOA). The models would need to be modified to account for other relationships, such as the U-shaped masking function that is common to metacontrast masking. However, the models should be easily modifiable to any dataset where phenomenal visibility can be theoretically related to a variable by some parametric function, simply by swapping out the function *s*(*x*), which in this case was a Weibull function, for the appropriate function.

## 5. Conclusion

In summary, we have shown that a difference in proportion correct thresholds between tasks requiring awareness and those that could be based on unconscious evidence is not necessarily the result of perception without awareness. In measuring perception without awareness, researchers need to be cautious of response bias, task differences and differences in stimuli. Our data showed a typical difference in thresholds that would normally be taken as evidence for perception without awareness; however, we were able to explain the pattern of performance with a model that assumed observers were making decisions based on the same perceptual evidence in all tasks. We found that although differences in thresholds were not the result of response bias, they were probably the result of a difference in task demands and possibly also a difference between the target stimuli's visibility. This result is not evidence against the concept of perception without awareness, but simply demonstrates that a difference in threshold does not guarantee perception without awareness. It is important for the study of conscious awareness that stimulus presentations can be categorically demonstrated to be processed with or without awareness. On the basis of the evidence presented in this paper it is recommended that patterns of behavioural performance suggestive of perception without awareness should be analysed to confirm whether the judgements could have been made on the basis of conscious evidence: stimulus processing should be assumed conscious until proven otherwise.

## Supplementary Material

Main Analysis

## Supplementary Material

'Conscious' models

## Supplementary Material

'Unconscious' models

## Data accessibility

Data and code have been attached as electronic supplementary material.
